# Online multidisciplinary treatment of binge eating disorder in people with high weight: a case series study

**DOI:** 10.1186/s40337-023-00809-9

**Published:** 2023-06-01

**Authors:** Mariana Valdez-Aguilar, Rosalia Vazquez-Arevalo, Xóchitl López-Aguilar, Ana Olivia Ruíz Martínez, Magda Rosinska, Juan Manuel Mancilla-Díaz

**Affiliations:** 1 Facultad de Estudios Superiores Iztacala, Universidad Nacional Autónoma de México, Estado de México, México; 2 Universidad Autónoma del Estado de México, Zumpango, Estado de México México; 3 Universidad Autónoma de Barcelona, Barcelona, Spain

**Keywords:** Feeding and eating disorders, Binge-eating disorder, Psychological intervention, Obesity

## Abstract

**Background:**

Binge eating disorder (BED) is the most frequent eating behavior among the general population (Guerdjikova in Med Clin 103:669–680, 2019). Many studies on interventions and BED treatments have been carried out in the United States and Europe, few have been reported in Latin American populations. People with this disorder not only have physical consequences of it but also social and psychological ones, therefore a multidisciplinary treatment approach is a good option to treat this condition.

**Objective:**

To evaluate the feasibility of a multidisciplinary online intervention (i.e., psychological, nutritional, and physical activity) in patients with BED.

**Method:**

The design was a case series study of two clinical treatment groups, with pre-test and post-test psychometric measures along with two follow-ups (at 2 and 6 months). Fifteen people diagnosed with BED (2 men and 13 women), with a mean age of 34.93 years (SD=11.91) and a mean initial BMI of 42, participated in this study. The treatment was carried out over the span of 28 sessions, each one being two hours per week consisting of 1 hour of group therapy and 1 hour of individual therapy. There were four evaluations: pre, post and two follow-ups.

**Results:**

Five patients did not complete the treatment (30%). The comparisons were made through the non-parametric Friedman test, finding a statistically significant decrease in binge eating symptoms (x2=15.57; *p*=.001), anxiety symptoms (x2=15.96; *p*=.001) and depression (x2=15.03; *p*=.002). There was an improvement in clarity (x2=11.60; *p*=.010) and emotional regulation (x2=7.75; *p*=.050), only in women. The patients reduced their body weight, and improved their eating and exercise habits by introducing fruits and vegetables and including 20-30 minutes of physical activity into their daily routine. Regarding the Objective Clinical Change Index, in terms of the objective clinical change, a positive change was observed in all the variables addressed.

**Conclusions:**

The data presented allowed us to conclude that the online multidisciplinary intervention was effective in the treatment of BED.

*Trial registration* Retrospectively registered

## Abstract (plain language summary)

Binge eating disorder (BED) is the most frequent eating disorder, however, it is underdiagnosed and undertreated, therefore it is currently considered a serious health problem. There are various psychological treatments for BED, but most of them focus on eating, not including the role played by emotions in this disorder. The importance of a multidisciplinary form of treatment (i.e., psychological, nutritional, and physical activity) has been observed in previous studies, emphasizing the importance of the psychological aspect which can improve treatment outcome. Hence the objective of this study was to evaluate the effectiveness of an online multidisciplinary intervention in patients with binge eating disorder. The treatment consisted of 28 two-hour sessions in which contents of psychoeducation, motivation, improvement of eating habits, emotional regulation, and modification of lifestyle in general were addressed. Assessment instruments were applied before and after treatment as well as at two follow-ups. Statistically, improvement was observed in all areas, in addition, these improvements were maintained in the follow-up evaluations at two and six months. The findings of this study are significant given that it is one of the first case series studies carried out in Mexico in this population. Therefore, it is important to continue research on this topic while also being able to provide a timely diagnosis and treatment for these patients.

## Background

Binge eating disorder (BED) is characterized by the consumption of large amounts of food, greater than those that would be normally eaten, in short intervals of time and accompanied by a sense of loss of control. It differs from Bulimia Nervosa (BN), since there are no compensatory behaviors (e.g., vomiting, purging, excessive exercise, use of diuretics and/or laxatives) [1]. In 1994, BED was proposed for research in the fourth version of the Diagnostic and Statistical Manual of Mental Disorders [2] and since May 2013, it is officially recognized by the American Psychiatric Association [3] as an eating disorder such as Anorexia Nervosa (AN) and BN. BED is the most common eating disorder [4] with a prevalence in community samples between 2 and 6% [5, 6] and in clinical samples, between 30 and 54% [7].

The causes of BED are complex. Binge eating episodes are related to negative feelings such as guilt and shame, and mood changes such as depression and anxiety [4, 8], as well as difficulties in emotional regulation and an association with stressful life events [9]. Given this, more attention is needed regarding the mental health of individuals with BED and further research regarding its treatment [10].

Psychological therapy is the first-choice intervention for BED. There are various psychotherapies such as Cognitive Behavioral Therapy (CBT, [11]) Interpersonal Therapy (IPT, [12]), Dialectical Behavioral Therapy (DBT, [13]) and Cognitive Affective Integrative Therapy [14] that have a similar efficacy [14]. However, CBT is the most widely used form of intervention, as it has proven to be an effective treatment for people with this disorder. CBT consists of psychoeducation; analyzing problems and goals; food intake planning, and modification of cognitive distortions related to food and weight [9].

Some studies [15–17] have observed that implementing a multidisciplinary intervention has greater benefits in both the short and long term [2, 16]. These programs have four main components: 1) psychological therapy, 2) nutritional recommendations, 3) physical activity, and 4) medical monitoring [17]. In the psychological component, patients are motivated to adhere to treatment with strategies such as self-monitoring during meals, setting realistic and achievable goals, reflecting on the origin of binge eating and the disorder, as well as being provided with alternative strategies and behaviors. The nutritional part of this therapy is generally designed to educate the individual about healthy eating habits and restructure the consumption, purchase, and organization of food. The physical component consists of simple daily exercises such as walking or dancing which may produce weight loss and enhance muscle mass. Finally, the medical component aids in monitoring medical conditions and/or comorbidities associated with BED [18].

On the other hand, due to the COVID-19 pandemic, a large part of mental health treatments, including eating disorder programs, have migrated to digital health platforms.

Previous research [19, 20] has reviewed the potential of online interventions for eating disorders. They found that these interventions have a high efficacy, and appear to be an improvement when compared to other programs (e.g., in reducing BED psychopathology and binge eating frequency), especially in individuals with fewer comorbidities. Given this reasoning, it is necessary to continue evaluating the contributions of online treatments, especially in patients with BED. Therefore, the objective of this study was to evaluate the feasibility of a multidisciplinary intervention (i.e., psychological, nutritional, and physical activity) online in patients with BED.

## Methods

### Ethical considerations

Approval of the study was granted in August 2020 by the UNAM Psychology Postgraduate Ethics Committee [reference number 0165] in Mexico City, Mexico. All the participants signed an informed consent document which stated the objective of the study, the participation requirements and adherence in this study. In addition, in accordance with the ethical principles of Helsinki, they were assured that the information provided was confidential and voluntary.

### Research design

An uncontrolled case series design was used to examine the evidence for an online multidisciplinary intervention. The treatment of this intervention combined both group and individual sessions. Outcomes were reported at assessment, prior to treatment, upon completion of treatment along with two follow-ups (2 and 6 months) [21].

First, a pilot study was carried out regarding this intervention. Then, some adjustments were made to the intervention and participants were recruited via the social communication page of UNAM FES Iztacala. Those interested in this type of treatment had to be people who were concerned with their way of eating. Initially, 150 people responded to the announcement via email. First, they were asked to answer two screening instruments: 1) Binge Eating Scale and [22] 2) Weight and Eating Patterns Questionnaire [23]. The participants who exceeded the cut-off point for both instruments were asked to answer the rest of the psychological battery, which was sent to them by email through google forms. Then, a diagnostic interview based in the DSM-5 [24] was scheduled, which was then carried out through the online platform Zoom, with the psychologists in charge of the project conducting the interview.

### Participants

Fifteen adults participated (2 men and 13 women), with a mean age of 34.93 years (SD=11.91), with an initial mean weight of 112 kilograms (kg) and a mean body mass index (BMI) of 41.73, which is categorized as Obesity Class II. The participants reported 4 or more episodes of bingeing per week. In regard to their socioeconomic & educational status, 73% completed university studies, 60% were single and 34% had a history of addiction (e.g., alcohol or smoking). Lastly, regarding medical comorbidities, 26% of the participants presented with polycystic ovary syndrome (see Table 1).

**Table 1:** Sociodemographic variables

	Participants (N = 15)	Frequency %; (SD)
Sex	Male	2 (13.3%)
	Female	13 (86.7%)
Age Range = 19–55 years		34.93 (11.91)
Weight		112 (31.86)
Body Mass Index (BMI)		42.01 (9.36)
	Obesity Class 1	5 (33.3%)
	Obesity Class 2	2 (13.3%)
	Obesity Class 3	8 (53.3%)
Binge eating episodes per week		4 (2.3)
Education	High School Diploma	2 (13.3%)
	Bachelor’s Degree	11 (73.3%)
	Master’s degree or higher	2 (13.3%)
Occupation	Student	5 (33.3%)
	Employed	6 (40%)
	Housewife	2 (13.3%)
	Unemployed	2 (13.3%)
Marital status	Single	9 (60%)
	Married	2 (13.3%)
	Divorced	4 (26.7%)
Addiction	Alcoholism	4 (26%)
	Tobacco	1 (6%)
	None	10 (66%)
Comorbidities	Hypothyroidism	2 (13.3%)
	Hyperthyroidism	1 (6%)
	Polycystic ovary syndrome	4 (26%)
	Pre-diabetes	1 (6%)
	None	7 (46%)

*SD* Standard deviation

### Inclusion and exclusion criteria

The inclusion criteria were that: 1) participants were adults (18 years or older), they volunteered to participate in the study and adhere to the proposed schedules, 2) they acknowledged that they had problems with eating, 2) the diagnostic interview resulted in a BED diagnosis according to the diagnostic criteria of the DSM-5.

The exclusion criteria were that they: 1) did not sign the informed consent document, 2) were already participating in another psychological, medical and/or nutritional treatment program and 3) had a psychiatric comorbidity (e.g., schizophrenia, bipolar disorder and/or risk of suicide) or medical (e.g., uncontrolled hypertension and heart and/or liver disease) that prevented them from participating in this study.

The elimination criteria were: 1) failure to attend more than two sessions and 2) failure to complete the evaluation follow-ups.

### Dropout during treatment

Of the initial 15 participants, 5 women abandoned treatment, representing 30% of the sample size. Two of the five people reported physical obstacles, 1 had to return to work in person and the other changed her place of residence. Regarding the other three, 2 people were asked to leave due to a lack of compliance to the treatment framework and 1 required psychiatric treatment.

### Diagnostic assessment

Clinical assessments took place before and after the intervention and at 2 and 6 months follow up. Group evaluation feedback forms were completed online at the final group session.

1. *Sociodemographic data questionnaire* Created for this research study, it consists of 8 questions regarding participant sociodemographic data.
2. *Daily self-registration* A self-registration document was developed to have data about daily food consumption, number of binge episodes, associated emotions, as well as physical activity performed each day.
3. *Interview for the Diagnosis of Eating Disorders* (IDED; [25]) It is a semi-structured interview consisting of 18 questions. Its purpose is to determine the differential diagnosis between AN, BN, and BED, with questions related to patient history, symptoms, and general evaluation items. The duration of this interview is up to 45 min. The Spanish version [24] of this interview was used for this study.
4. *Binge Eating Scale* (BES [26]) This scale is used to detect the presence of BED as well as the intensity of binge eating, from moderate to severe [4]. This scale was validated for Mexico [22] and had a recent confirmatory analysis by Valdez-Aguilar [22]. This scale has 16 items, consisting of 2 dimensions, cognitive and behavioral manifestations. It was found that Cohen’s alpha showed good internal consistency in the Mexican validation (α = 0.88 for cognitive manifestation and α = 0.87 for behavior manifestation).
5. *Questionnaire on Eating and Weight Patterns* (QEWP-R [27]) It is used to identify individuals with recurrent binge eating episodes with a feeling of loss of control and guilt, in the absence of inappropriate compensatory behaviors typical of BN, according to the DSM-5. The questionnaire consists of 13 items, which are about the amount of food consumed during the binge, its duration, and feelings of guilt regarding food, diet, and weight. It was validated in Mexico and showed good reliability and validity [23].
6. *Trait Meta-Mood Scale-24* (TMMS-24) There is a Spanish version [28]. It assesses meta-knowledge of emotional states. It consists of 24 items on a Likert-type scale and is organized into three factors: 1. Attention 2. Clarity or Comprehension and 3. Regulation of emotions.
7. *Beck Anxiety Inventory* (BAI [29]) It contains 21 questions that evaluate anxiety symptoms and their severity. The Mexican version of the BAI [30] presents a high internal consistency of 0.84 and 0.83, in students and adults respectively. The qualification norms in the Mexican population are from 0 to 5 points signifying minimum anxiety; from 6 to 15, mild anxiety; from 16 to 30 points, moderate anxiety; and from 31 to 63, severe anxiety. The criterion to consider anxiety as clinically relevant is to obtain 16 or more points.
8. *Beck Depression Inventory* (BDI [31]) It contains 21 items that evaluate symptoms of depression and its severity, for the Mexican population it was translated, adapted, and validated showing good properties The higher the score, the greater the severity of depressive symptoms [32]. A score greater than 10 reveals the presence of depression. This instrument has adequate concurrent validity (r = 0.70) and high internal consistency (α = 0.87).

### Pilot study

Before carrying out the main study, a pilot test was carried out with 4 participants (3 women and 1 man) to evaluate each session, the topics, content, form of application and make the pertinent changes and adjustments. The pilot study consisted of 24 face-to-face sessions. In this study, the sessions were carried out individually. Some topics were addressed in conjunction with a nutritionist and a sports doctor. The nutritionist participated in the first module of the intervention, that is, the first two sessions, and in the last module in order to reinforce some concepts; teaching the participants to regulate their diet, create healthy menus and plan their meals, in addition, at each session the participants were weighed and measured. The sports doctor gave them a session on the physical consequences of obesity, as well as the benefits of physical activity in daily life.

### Therapeutic intervention

The multidisciplinary intervention was completed in three phases: 1) Motivation for treatment and psychoeducation about BED. 2) Work on different clinical topics and 3) Relapse prevention. Two treatment groups were formed according to the level of severity of the disorder, considering the number of binge eating episodes and the history of crucial life events. All treatment sessions were conducted via Zoom. From the initial session they were asked to start recording their daily meals and binge eating in the self-report document. In this way, 10 patients completed the 28 sessions of the multidisciplinary intervention and the follow- ups. Table 2 shows the phases and themes of the entire multidisciplinary treatment. Five psychologists, 2 nutritionists and a sports physician participated in this intervention. The entire team participated in the sessions according to their area of expertise

**Table 2:** Multidisciplinary treatment sessions

Treatment phases	Session	Objectives
Motivation for treatment	1. Motivation and Psychoeducation	Give psychoeducation about the disorder and an overview of the treatment is disclosed
	2. Taking responsibility for my emotions	Introduce emotions and how to manage them in different situations
	3. Healthy Eating	Eat in a planned and structured way to avoid the restriction-binge cycle
	4. Planning my diet	Restructure the purchase and organization of food
	5. Who am I?	Understand the importance of knowing themselves
Work on different clinical topics	6. Physical activity	Know the importance of physical activity in daily life
	7. Freeing myself from my past	Work with emotions and situations from the past
	8. Self-esteem	Work with self-esteem and make the participant realize their strengths
	9. Strengths and weaknesses	Work with self-esteem and make the participant realize their strengths and weaknesses
	10. Body image (Part 1)	Work with the positive aspects of the participants and beauty standards
	11. Body image (Part 2)	Work on reconciling with themselves and accepting their own body
	12. Physical activity	Monitor physical activity and exercises performed at home
	13. Petitions and conflict resolution	Solve problems and conflicts assertively
	14. Who am I and where am I going?	Review the tools that have been used throughout the treatment
	15. Emotional regulation	Know and use emotional regulation strategies
	16. Being a man and being a woman	Analyze the role of being a man and being a woman today
	17. Evaluating my body weight	Review weight charts for each participant throughout the treatment
	18. Griefs	Make known what a grief is and its process
	19. Loss and grief	Work on the experience of loss and its elaboration
	20. Goodbyes	Explain the process of mourning and their individual work
	21. Emotional wounds (Part 1)	Know emotional wounds and their difference from emotional trauma
	22. Emotional wounds (Part 2)	Know emotional wounds and their difference from emotional trauma
	23. Resilience	Analyze what resilience is and its application in different life situations
	24. Maternity and paternity	Explain the relationship of parents with children through motherhood and fatherhood
Relapse prevention	25. Relapse prevention	Explain what a fall and relapse is and ways to deal with them
	26. A new lifestyle	Develop a life plan with the tools reviewed in the treatment
	27. Preparing for my future	Carry out a closure plan and follow-up of what was reviewed in the treatment
	28. New beginnings	Finalize treatment

### Format of the intervention

The treatment was carried out over 28 sessions for seven months with a frequency of 1 weekly meeting. Each session lasted two hours. The first hour consisted of group work and the second consisted of an individual session. The sessions always began with a review of the daily self-records by a nutritionist, then feedback was given about the nutritional goals set at the beginning of the treatment and the planning of meals and portions was reviewed. After this nutritional feedback, which usually lasted about 20 minutes, the topic to be reviewed during the session was presented. The topic was presented in a psychoeducational format while generating a group reflection on it, this portion lasted 30 minutes. Then there was a 10-minute period where the participants performed simple exercises to encourage physical activity.

### Individual sessions

The second hour of the session consisted of a one-on-one session with a therapist lasting 45-60 minutes. In this part the participants discussed the topics from the previous hour along with emotional and life events related to BED or binge eating. Individual sessions were used to problem solve idiosyncratic concerns that arose as the treatment progressed that were not fully addressed in the group session.

### Treatment fidelity

All therapists had been trained and were experienced in delivering the multidisciplinary treatment. The entire team met regularly to assure adherence to the original format for both the group and individual intervention as well as to share ongoing practice and supervisory reflections and knowledge.

### Data analysis

Given the small number of participants and due to the standardization of treatment, it was decided to analyze the two groups as one. Nonparametric tests were used, the Wilcoxon rank sum test was used to compare before and after differences within the group. And to observe the changes in the measures of the pre-test, post-test and follow-ups, the Friedman test was used. In addition, an objective clinical change analysis was performed to assess changes at the individual level. All data was analyzed through SPSS version 27.

## Results

### Binge eating symptomatology

Table 3 shows the results of the psychological variables from the pre-assessment to the second follow-up. Regarding eating symptoms, statistically significant differences were found, both in the total score of the BES (x2=15.57; *p*=.001), and in the QEWP-5 (x2=13.58; *p*=.004), observing a significant decrease in binge eating and, in the symptoms, associated with the disorder.

**Table 3:** Comparisons of the means of all the participants through the Friedman test between the baseline and the follow-ups in the clinical variables of the multidisciplinary treatment

			N = 10				
Scales	Gender	Pre	Post	1st follow-up	2nd follow-up	X	P
BES		32.13 (4.22)	7.55 (9.33)	9.12 (6.91)	9.12 (4.79)	15.57	.001*
QEWP-R		8.38 (1.92)	3.50 (2.32)	4.75 (2.76)	4.37 (3.02)	13.58	.004*
BAI		31.75 (10.20)	11.75 (12.25)	15.12 (10.56)	12.62 (5.75)	15.96	.001*
BDI		24.12 (8.95)	7.37 (12.08)	9.00 (7.81)	5.37 (3.29)	15.03	.002*
TMMS-24							
Atention	Female	28.00 (7.97)	25.14 (6.14)	25.75 (6.04)	26.87 (6.92)	2.72	.430
	Male	29.00 (1.41)	26.00 (5.65)	27.00 (5.65)	27.31 (6.08)	1.31	.360
Clarity	Female	21.57 (7.45)	29.75 (7.22)	26.75 (6.20)	29.25 (7.00)	11.60	.010*
	Male	23.50 (0.70)	21.50 (2.12)	29.50 (7.77)	29.75 (6.00)	4.10	.444
Regulation	Female	22.00 (5.44)	30.72 (6.75)	27.37 (6.86)	26.75 (5.80)	7.75	.050*
	Male	28.00 (7.07)	28.50 (6.36)	24.00 (7.07)	24.75 (5.14)	0.28	.860

*BES* Binge eating scale, *QEWPR* Questionnaire on eating and weight patterns revised, *BAI* Beck Anxiety Inventory, *BDI* Beck Depression Inventory, *TMMS-24* Trait meta-mood scale-24. Only the TMMS-24 is presented by sex because it is measured in both categories

Bold value indicated the difference is significant at 0.05 level

^*^p < 0.05

### Symptoms of anxiety and depression

For anxiety symptomatology, significant differences were found from the pre- evaluation to the second follow-up (x2=15.96.96; *p*=.001), based on the total scores measured through the BAI questionnaire. It was observed that at the beginning the participants presented an average score (X= 31.75, SD=10.20) that indicated moderate anxiety and at the end of the treatment (X=11.75, SD=12.25) they showed no symptoms, which was maintained in the follow-ups. Regarding the depression symptomatology which was measured with the BDI total score, statistically significant differences were present (x2=15.03; *p*=.002). At the beginning, participants presented moderate depressive symptoms (X= 24.12, SD=8.95) and at the end they did not present with any depressive symptoms (X=7.37, SD=12.08), this was maintained throughout the follow-ups.

### Emotional intelligence

Regarding the emotional intelligence results, which were measured through the TMMS-24, statistically significant differences were found from the pre-evaluation to the second follow-up in the factors of clarity (x2=11.60; *p*=.010) and emotional regulation (x2 =7.75; *p*=.050) only in women. Thus, female participants improved their understanding and emotional regulation, while no changes were observed in men via their TMMS-24 scores. However, this did not correspond to what was observed during the sessions, where they presented with a lack of emotional expression. This lack did not translate in the self-assessment of their emotions, therefore their scores did not change throughout the treatment (see Table 3). Therefore, although they mentioned an improvement at the end of the intervention, this was not reflected in the instrument, as their scores were already adequate from the beginning.

### Body weight, diet, and physical activity

#### Body weight

Regarding body weight, a reduction was observed from the pre-evaluation to the second follow-up, this ranged between 3 and 15kg, with women having the greatest decrease. Additionally, in the first follow-up, most of the participants maintained the same weight as at the end of treatment, however, there were those who continued to lose weight, observing a reduction of up to an additional 4kg. At the second follow-up, all participants maintained their weight loss.

#### Eating habits

At the beginning of treatment, little consumption of fruits and vegetables was observed, especially at breakfast, in addition, the consumption of sugars, cereals and tubers was high, this was observed especially at night, consisting of 4 to 6 times a week. After the intervention, a decrease was observed, from 2 to 3 times per week, in terms of the consumption of sugars. In addition, an increase in the consumption of vegetables and fruits was constant, meaning there was a daily consumption.

#### Physical activity

At the beginning of the intervention, two of the participants performed exercise or some other recreational activity sporadically. Three of the participants had a childhood history of doing some exercise aimed at losing weight. After treatment, all participants were able to adhere to physical activity in their daily routine, performing it on average two to seven times a week with low to medium intensity activities. During the follow-ups, it was observed that the frequency of the activity decreased, as the patients reported doing it between two and three times a week. Among the activities they began to perform were walking, yoga, cycling and cardio. Some patients reported that the physical activity had an impact on their emotions, mentioning feelings of increased motivation to carry out their daily activities.

In the case of males, it should be noted that performing physical activities was more difficult, even though one of them had a history of playing sports in childhood. However, it was possible for them to increase their activity from two to three times a week. In females, dance was noted to be a motivator for them to be physically active, doing it at home with videos and YouTube routines, supervised by the sports physician.

### Objective clinical change index of the participants

To find out the differences at the individual level after the multidisciplinary intervention ended, each case was analyzed obtaining the Objective Clinical Change Index (OCC). A significant clinical change is considered when it is greater than 20%; it is necessary to consider that, depending on the direction of the data, it may indicate clinical deterioration or improvement [33].

All the participants obtained a positive change greater than 65% in binge eating symptoms, in terms of anxiety symptoms there were also changes between 40 and 90% in the participants. In the same vein, depression symptoms decreased from 88 to 72%. Regarding emotional intelligence, there were positive changes in six patients in the attention factor between 27 and 44%. Likewise, in emotional clarity, changes were observed in eight participants between 47 and 100%. And lastly in emotional regulation there were positive changes in eight patients between 21 and 100%.

## Discussion

The main objective of this study was to evaluate the feasibility of a multidisciplinary online intervention in a group of clinical case series patients with BED. Considering that most of the participants were women, we can say that, according to the literature, women are more likely to seek help for an eating disorder even though there are similar rates of BED by sex [5]. In addition, it has been observed that in men there is less awareness of eating psychopathologies and greater reluctance to seek treatment for a disorder that they possibly consider "feminine"[7]. Given this, it was important for patients to decide to seek treatment, as many of them were unaware of BED and how the disorder influenced their emotions and eating habits.

It is important to mention that there were men in this study and that they were the ones who presented the greatest emotional and eating psychopathology. Thus, their incorporation into more treatment programs is significant since, even though BED is commonly reported in men (2.5 % in men vs. 3.0% in women), they are underrepresented in clinical treatment studies [15]. In this sense, two community studies have found few differences in physical and psychosocial deterioration according to sex, in relation to binge eating symptoms [34, 35]. Therefore, it is necessary to continue implementing strategies to ensure that more men attend and remain throughout the treatment process. In our study, the two men were able to complete the treatment, observing good effectiveness and an improvement in their quality of life.

On the other hand, it is also necessary to point out that there was a dropout of 30% of the participants during treatment. According to a review [36], the average online treatment dropout is 29%, so our study was within this mean. It has been shown that by measuring patient adherence (e.g., participation in the program, attention given to the time spent in a session, written exchange with the therapist, submission of assignments) it is possible to prevent dropouts in Internet-based programs for BED. Therefore, adherence should be monitored and encouraged during the program [37]. Additionally, in future research it would be important to consider the potential dropout rate of the participants.

Patients at the beginning of treatment showed severe symptoms and subsequently a positive clinical change from 94 to 67%, which lasted until the second follow-up. This result is important because it indicates that a central aspect of BED decreased and is consistent with two studies which are considered to be effective, that were also conducted online. The first one being CBT based, which significantly reduced binge eating episodes [38] and the second saw a reduction in the frequency of binge eating lasting throughout the follow-ups carried out [20]. In both studies food records were also used and the therapeutic relationship, between the patient and therapist, was important for the patients to be motivated to continue treatment.

Regarding symptoms of depression and anxiety at the start of treatment, a moderate trend was observed. These findings are consistent with other studies that show that more than half of the patients with BED who complete online treatment suffer from symptoms of anxiety and depression. Above all, it has been observed that depression is a comorbidity that is recurrent in men with eating disorders [39]. Both the symptomatology of anxiety and depression are aspects that have been pointed out in the literature as clinical symptoms in BED. In this sense, it was encouraging to observe that the significant decreases in these clinical variables were maintained throughout the follow-ups.

Regarding emotional intelligence, it was observed that there were improvements in the factors of clarity and emotional regulation only in women, while the emotional attention variable had no changes since both women and men showed adequate scores from the beginning. Some authors [40, 41] have provided evidence that people with BED are less able to manage emotions or use more dysfunctional regulation strategies such as repressing emotions or using binge eating as a maladaptive emotional strategy in the face of stressful social situations. Also, in men it has been observed that there is difficulty recognizing emotions [6]. Given the above information, our male patients exhibited limitations in emotional self- awareness from the beginning by not answering the instruments adequately, so it was not possible to quantitatively measure their progress.

Regarding body weight in the study participants, there was a reduction between 3 and 15 kilograms. It is important to note that the main objective of this treatment was not only the reduction of body weight, but moreover the resolution of eating problems for each patient. This primary aim had a clear impact on their body weight, due to the lower frequency of binge eating, which appears promising. The decrease and maintenance of body weight as a point of effectiveness for BED treatments is a point of discussion, since an improvement in quality of life and associated psychological variables has been observed in some interventions, without a decrease in body weight. Recently, it has been examined that in multidisciplinary formats, that is, by combining the psychological, nutritional, and physical activity parts, there is a reduction in body weight and also a greater efficacy in improving eating psychopathology [42, 43].

Regarding food, it was initially observed that the participants consumed many grains, fried foods and sugars and there were no fruits or vegetables at home. After the intervention, the intake of fried foods and sugars decreased and there was an introduction of vegetables and fruits, some patients were even involved in the preparation of food at home. It is important that patients with BED learn to eat and be able to vary their diet, so the inclusion of the nutritionist in this study was essential. A review of the literature highlighted that a multidisciplinary intervention produced a greater impact in the treatment since it helps participants have a greater variety of meals and an improvement in binge eating symptoms compared to interventions that are only psychological or dietary [44]. This multidisciplinary approach was used, which had a notable impact on the improvement in patients in their way of eating as well as in their relationship with food and their quality of life.

In relation to physical activity at the beginning of the treatment, the patients did not engae in any physical activity, at the end of the intervention we observed that they introduced it three to four times a week. In this regard, physical activity interventions have shown promise for improving physical health [45], eating symptoms, such as decreased binge eating, as well as improved mood [46]. In our online treatment, it was observed that physical activity had an impact on both the diet and the emotional aspect of the well-being of each patient.

Finally, more evidence is needed to evaluate this type of online intervention in multidisciplinary formats, comparing them with face-to-face interventions. As well as to study their effects on participants and to evaluate the changes when performing the treatment virtually, adding technological applications and other components that could facilitate the intervention. In addition, it is necessary to guide health professionals so that these online programs are more effective and can be replicated both in the clinical field and in research.

The findings of the present study have some limitations. Regarding dropouts, more monitoring of the patients was needed throughout the treatment to assess the motivation for their participation in the intervention and to be able to prevent dropouts. On the other hand, the number of participants was small, resulting in limited power to generalize our results. Another limitation was that the findings in the emotional intelligence variable were not as efficient as expected, especially in men, since it was not possible to measure their progress throughout treatment, so evaluating changes was difficult. This is an area of improvement in future interventions given the important role that emotions play in BED. Another limitation was not being able to measure the patients anthropometrically and only having weight and BMI as reference, since these are measurements that may have restricted us from observing whether there was a greater impact on the participants, both in physical and nutritional health.

Among the strengths of the study, to the best of our knowledge, this is one of the first online multidisciplinary studies conducted with patients with BED in Mexico. This study takes a step forward in evaluating the efficacy of online multidisciplinary formats for patients with this psychopathology. In addition, the use of technology allowed the participants to have a good adherence to the treatment, helping in the performance of therapeutic tasks. Likewise, working in a collaborative team allowed for possibility to address various problems of the disorder and deepen its treatment. Finally, the follow-up periods helped to obtain more information and evidence on the medium and long-term effects of the intervention.

## Conclusions

This multidisciplinary treatment proved to be feasible and effective on the proposed clinical objectives: binge eating symptoms, anxiety and depression symptoms, improvement in emotional intelligence, eating habits, body weight and physical activity. The findings of the intervention are significant since it is one of the first online BED case series carried out in Mexico, so it is important to disseminate and monitor it. Future research is needed to confirm the effectiveness and mechanisms of change of this multidisciplinary treatment.

## Data Availability

The datasets used and/or analysed during the current study are available from the corresponding author on reasonable request.

## Abbreviations

AN: Anorexia nervosa
APA: American Psychiatric Association
BAI: Beck Anxiety Inventory
BDI: Beck Depression Inventory
BED: Binge eating disorder
BES: Binge eating scale
BMI: Body mass index
BN: Bulimia nervosa
CBT: Cognitive behavioral therapy
COVID-19: Coronavirus disease 2019
DBT: Dialectical behavioral therapy
DSM: Diagnostic and statistical manual of mental disorders
IDED: Interview for the diagnosis of eating disorders
IPT: Interpersonal therapy
OCC: Objective clinical change
QEWP-5: Questionnaire on eating and weight patterns-5
SD: Standard deviation
TMMS-24: Trait meta-mood scale-24
